# White noise insole: an artificial evoked sensation device that can be expected to improve plantar sensation of diabetic foot

**DOI:** 10.1038/s41598-023-47263-w

**Published:** 2023-11-24

**Authors:** Yangzheng Jiang

**Affiliations:** 1 Women’s Hospital, Zhejiang University School of Medicine, Xueshi Rd. 1, Hangzhou, China; 2 Institute of Rehabilitation Engineering and Technology, University of Shanghai for Science and Technology, Jungong Rd. 516, Shanghai, China

**Keywords:** Health care, Biomedical engineering, Mechanical engineering

## Abstract

Diabetic foot is a common severe complication of diabetes, and its main symptom is diabetic foot ulcer. The production of plantar diabetic foot ulcers is usually affected by two factors, namely neuropathy or vascular disease. While previous studies proved that stochastic resonance (SR) could effectively enhance the plantar touch of patients with diabetic feet, the potential impact of SR on neural circuit feedback, especially on the input of the tactile nerves of the lower limbs, is less clear. This study aims to explore the potential impact on the tactile threshold of the human foot when using vibrating insoles. We study a white noise vibration insole based on SR mechanism. We compare and analyze the tactile threshold voltage (TTV) triggered by an electrical stimulation device in three main plantar pressure-bearing areas (the second metatarsal (M2), the fourth metatarsal (M4), and the heel (H) area) of 8 participants using EEG and self-developed vibration insole. Significance found in M2 and M4 areas, white noise signal (WNS) lowered the tactile threshold in these areas, and had a potentially positive impact on patients with diabetic feet, especially in the M4 area. The influence of WNS on the plantar heel area was still controversial. This study showed that WNS applied to the sole could improve the plantar tactile sensing ability of patients with diabetic feet, but it did not cover all areas. The application of WNS showed better benefits for the forefoot area than for the hindfoot area, which was speculated that may be related to the difference in the distribution density of blood vessels in plantar areas. Due to the impaired natural touch in participants with diabetic foot, using artificial evoked sensation WNS intervention, would be a feasible approach to improve plantar sensation.

## Introduction

The foot is an essential human sensory organ, which plays an indispensable role in the sensory input of the body during standing and walking^1,2^. When the plantar tactile threshold is within an appropriate range, the sensory feedback at the plantar is in a steady-state and maintains the dynamic balance of gait movement^3^.

For patients with diabetic feet, due to plantar neuropathy or vascular disease, the plantar sensation will degenerate, and abnormal sensory feedback forces them to adopt compensatory walking strategies that increase fatigue^4^. Lack of natural sensory feedback significantly increases the risk of foot ulcers, and even worse, patients may suffer from lower limb amputation^5^. Therefore, compared with invasive surgical treatment, non-invasive and wearable technology may be a feasible solution to prevent and delay the progress of diabetic foot^6^.

As the positive link between stochastic resonance (SR) and biofeedback is revealed, it becomes possible to use engineering method to restore touch^7^. SR could be described a phenomenon that the presence of internal or external noise in a nonlinear system can increase the response of the system output^8^. At present, relevant SR phenomena have been observed in many physiological systems of human body, including the sensation system, such as touch, hearing and vision^9^.

The human body is a typical nonlinear dynamic system, and the application of subsensory noise can enhance the response of the nonlinear system to small signals^10,11^. The white noise signal (WNS) with constant power spectral density is one of the common sub-sensory noises, it has also been confirmed in previous study that WNS could increase the intensity of stimuli below the tactile threshold, thus achieving the effect of enhancing tactile sensation^11^. Further research indicates that WNS below the tactile threshold can significantly improve the sensory feedback of tactile impaired people, thus helping patients with diabetic reduce the risk of diabetic foot ulcers, restore them to normal gait and maintain balance^12^. While many previous studies proved that SR enhance the sensation^7^, the potential impact of SR on neural circuit feedback, especially on the input of the tactile nerves of the lower limbs, is less clear.

Therefore, the goal of this study was to design a white noise vibration insole based on the SR mechanism and verify its effectiveness through electrical stimulation experiment, which aims to explore the potential impact between the tactile threshold of foot and white noise vibration. It was hypothesized that the tactile threshold of the foot could be reduced under the intervention of white noise vibration.

## Materials and methods

### Equipment

**Figure 1:**
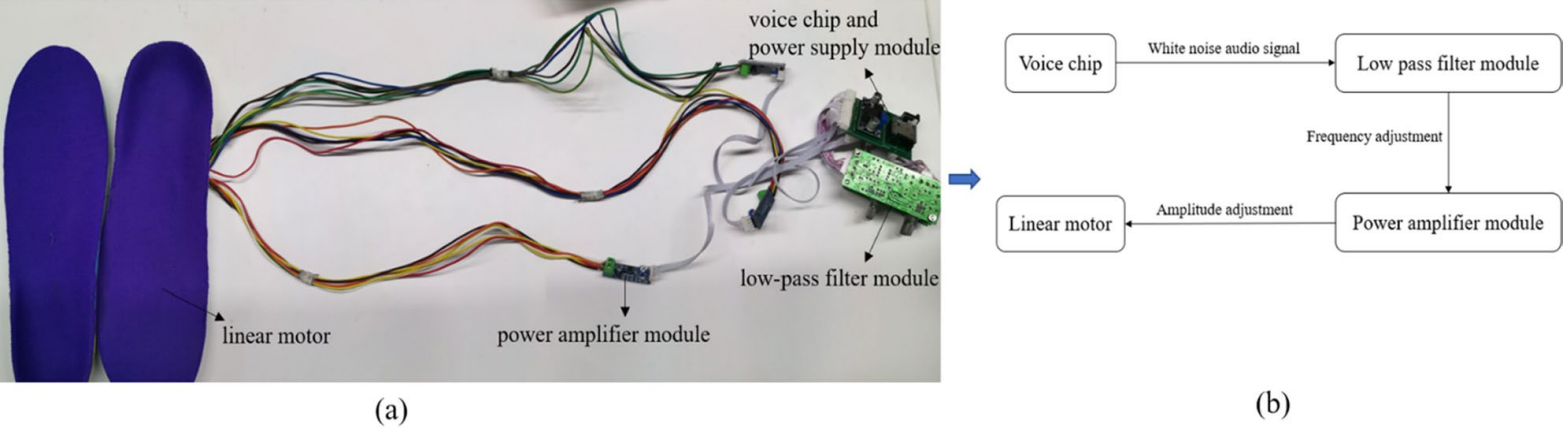
(**a**) White noise vibration insole. (**b**) The schematic diagram of the white noise vibration insole.

Our present study designed a white noise vibration insole for electrical stimulation experiments. As shown in Fig. 1a, this device mainly included five main parts: a voice chip, a power supply module, a low-pass filter module, a power amplifier module, and 3 linear motors. Figure 1b shows the schematic diagram of the white noise vibration insole. The selected white noise audio signal was stored in the voice chip. The audio signal could be filtered by a low-pass filter module before determining the appropriate frequency range. The filtered signal passed through the power amplifier module to adjust the amplitude of the signal, and then were transmitted to the linear motor. A total of 3 groups of linear motors (three per group) were embedded in the middle of the double-layer HI-POLY insole, and located at the second metatarsal (M2) area, the fourth metatarsal (M4) area, and the heel (H) area of the plantar anatomical landmarks, respectively. The reason for this design was that the above three areas were the main plantar pressure-bearing parts in daily walking^13^, the pressure in the M2, M4 and H areas was the highest of the foot, and the contact area with the ground in gait process accounts for a large proportion^14^. The low-pass filtered WNS was transmitted to the linear motor device, and the cutoff frequency was selected as 120 Hz, the device was switched by a controller and powered by a 12v battery. In addition, the equipment was calibrated with a multimeter to ensure that the vibration frequency of this device was stable.

**Figure 2:**
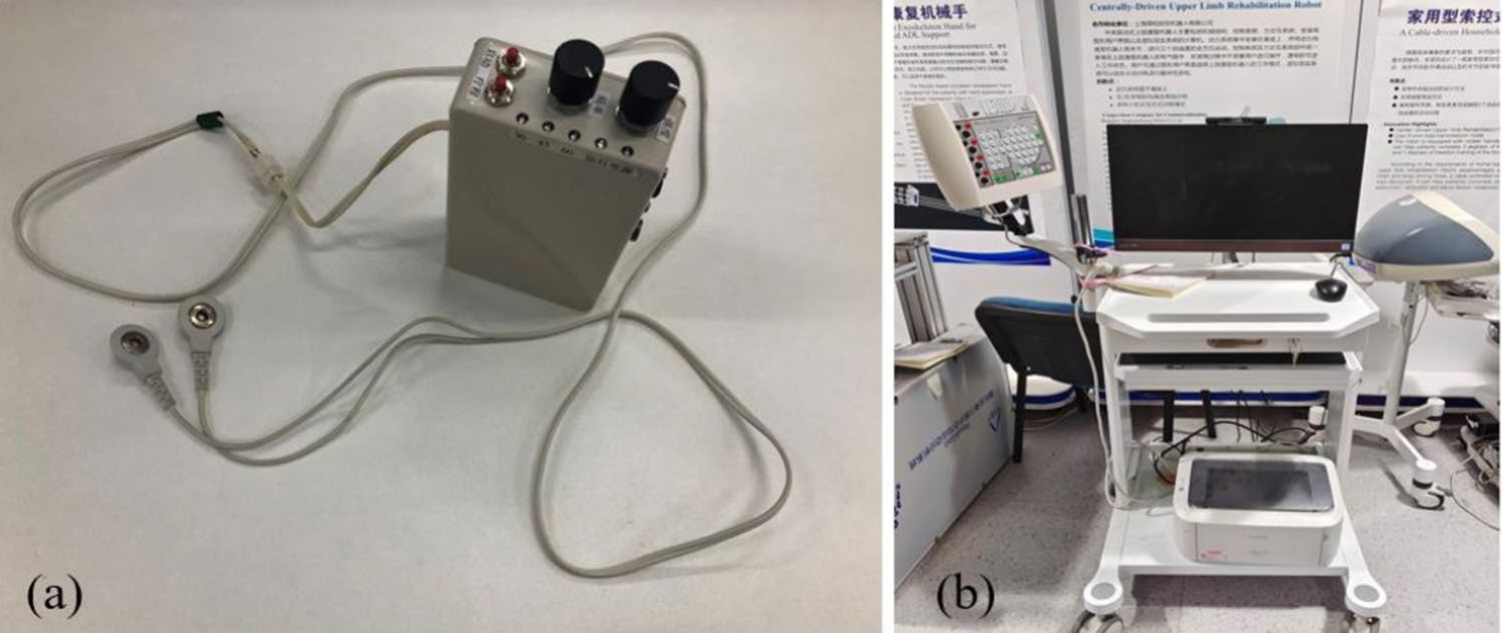
(**a**) Self- developed electrical impulse device. (**b**) EEG signal acquisition device.

A self-developed electrical impulse device was used for plantar electrical stimulation experiments, combined with the transient stimulus events generated by Electroencephalography (EEG), to objectively measure the tactile threshold voltage (TTV) of the first electrical impulse induced by participants. As shown in Fig. 2a, the device was equipped with two dry electrodes placed on the skin surface. The type of this device was electro cutaneous, which transmitted the current to the skin through the electrode, and stimulated the sensory nerve terminals by applying the current on the skin surface. The optional frequency of the device was 1–100 Hz, and the electric pulse voltage range was 0–10 V. To reduce the variability of the experiment, the frequency of electric stimulation was generally set to 30HZ so that participants could feel the tapping sensation rather than numbness^15^. Also, electrical stimulation activated nerve fibers and receptors in superficial and deep skin layers, which was precisely related to the neuropathy of participants in EG^16^.

The SynAmps2 system (Neuroscan, El Paso, Texas, USA) was used for the acquisition of EEG signals, as shown in Fig. 2b, EEG was more sensitive detection method when the body sensed stimulation^17^. In this study, the sampling rate of EEG data was set to 1000 Hz, and the passband was set to DC100Hz. Since this study required electrical stimulation of participants’ surface skin to simulate the changes of tactile sensation, in order to accurately obtain the tactile threshold of participants, we used the SynAmps2 system to measure the C3 area of the left cerebral cortex, namely the tactile area of the cerebral cortex^18^. Somatosensory Evoked Potentials (SEP) and EEG showed a positive correlation, and their amplitude and waveform characteristics usually vary due to various factors. For tactile stimulation, the amplitude of SEP usually varies between 1 and 20 microvolts^19^, and according to the normal distribution, we set the amplitude threshold at 10 microvolts in the experiment.

### Subjects and methods

We used G*power version 3.1.9.2 (University of Kiel, Kiel, Germany) to estimate a priori sample size, with a power of 80%, and an α level of 0.05. The expected effect size was calculated by the average value (3.27, 4.64) and similar standard deviation (0.28, 0.39) of TTV of M2 area in CG and EG. The result revealed that the sample size of 8 were sufficient for analysis.

The criteria for entry into the study were: history of diabetes mellitus (Type 2), 45 ≤ age ≤ 65 years and presence of peripheral sensory–motor neuropathy. Finally, a total of eight participants with diabetic foot volunteered for this study as experimental group (EG). Besides, we recruited eight healthy participants as control group (CG). All participants signed informed consent before the start of the experiment. All methods were performed in accordance with the Declaration of Helsinki. The demographic data of participants are summarized in Table 1.

**Table 1:** Demographic data of participants.

Physical characteristics of participants	CG	EG
N (male/female)	7/1	7/1
Height (cm)	168.6 (3.0)	167.9 (5.5)
Age (years)	56.5 (7.8)	58.4 (5.7)
Foot length (cm)	25.2 (0.8)	25.4 (0.6)
Ankle width (cm)	8.3 (0.9)	8.5 (0.8)
Body weight (kg)	80.1 (9.8)	82.4.0 (10.9)
Duration diabetes mellitus (years)	–	13.0(5.8)
HbA1c (%)	4.5(0.8)	8.2(1.2)

Values are expressed as mean ± SD.

To avoid the contribution of the white noise vibration insole to baseline EEG, we had conducted baseline EEG tests in healthy participants, and the results showed high consistency of EEG baseline: our white noise vibration would not exceed the participant’s threshold, regardless of whether the white noise is turned on or off in the experiment. Therefore, the white noise vibration in our experimental conditions would not affect the baseline EEG.

**Figure 3:**
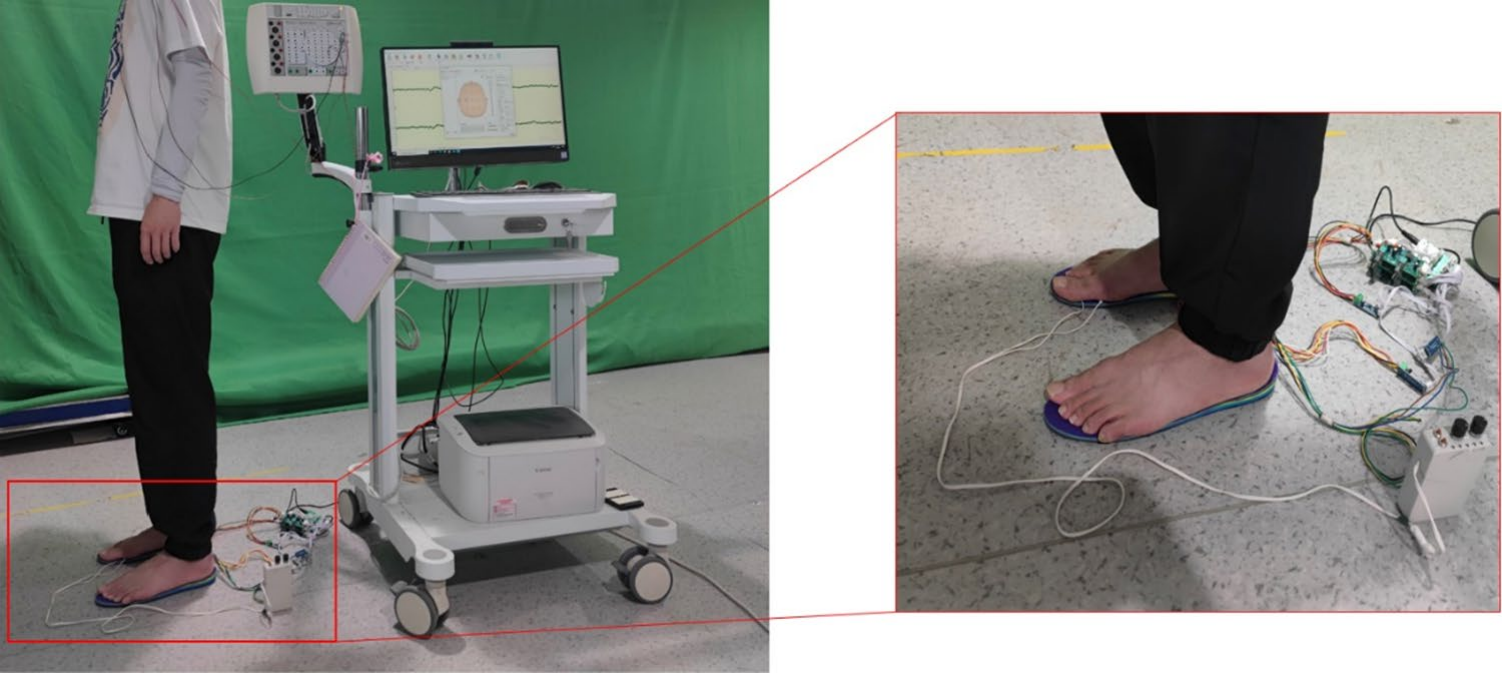
Schematic diagram of experimental setup: Participants stood stationary on a white noise vibration device, assisted by an electric pulse device and EEG for measurement.

**Figure 4:**
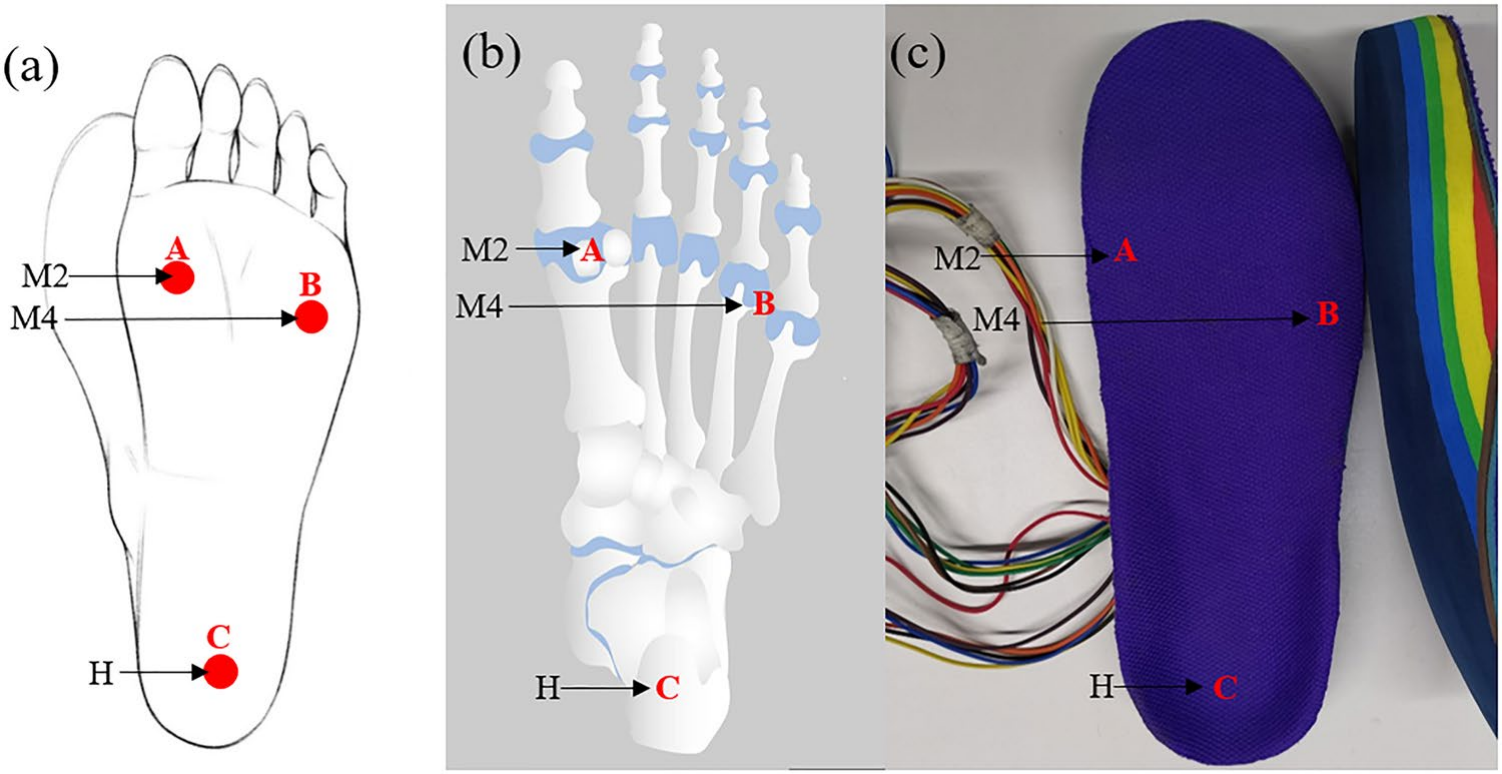
(**a**) Three placement positions of dry electrode patches for electrical impulse device. (**b**) Anatomical location of M2(A), M4(B), and H(C) areas. (**c**) Positioning of M2(A), M4(B), and M5(C) areas corresponding to insoles.

Each participant was required to participate in two groups of experiments, each group of tasks for each participant needs to be repeated three times. As shown in Fig. 3, participants were asked to stand still with their eyes closed on the white noise vibration insole (open or closed state), we sequentially conducted continuous electrical stimulation on the three anatomical landmarks M2, M4 and H areas of participants’ foot, then gradually adjusted the voltage of the electric impulse device to increase the intensity of electrical stimulation. When participants sensed the minimum stimulus, EEG would quickly fluctuate, the plantar tactile receptors were first stimulated by effective responses. Finally, we recorded the voltage of the electrical stimulation device at this time, namely the tactile threshold voltage (TTV). The conditions (white noise and control) were presented in random order to avoid biases in the results. Each intensity of electrical stimulation lasts for 5 s and the gap of the rise for each electric stimulation was set to 0.1v, range from 2 to 6 v. The 5-s duration was often longer than the time it took for the participants to perceive the stimuli and made subjective response^20^. This set also ensured an adequate recording of experimental data and minimized the occurrence of biases. The relevant positioning of the experiment was shown in Fig. 4. The tasks were performed indoors, on the same level.

**Figure 5:**
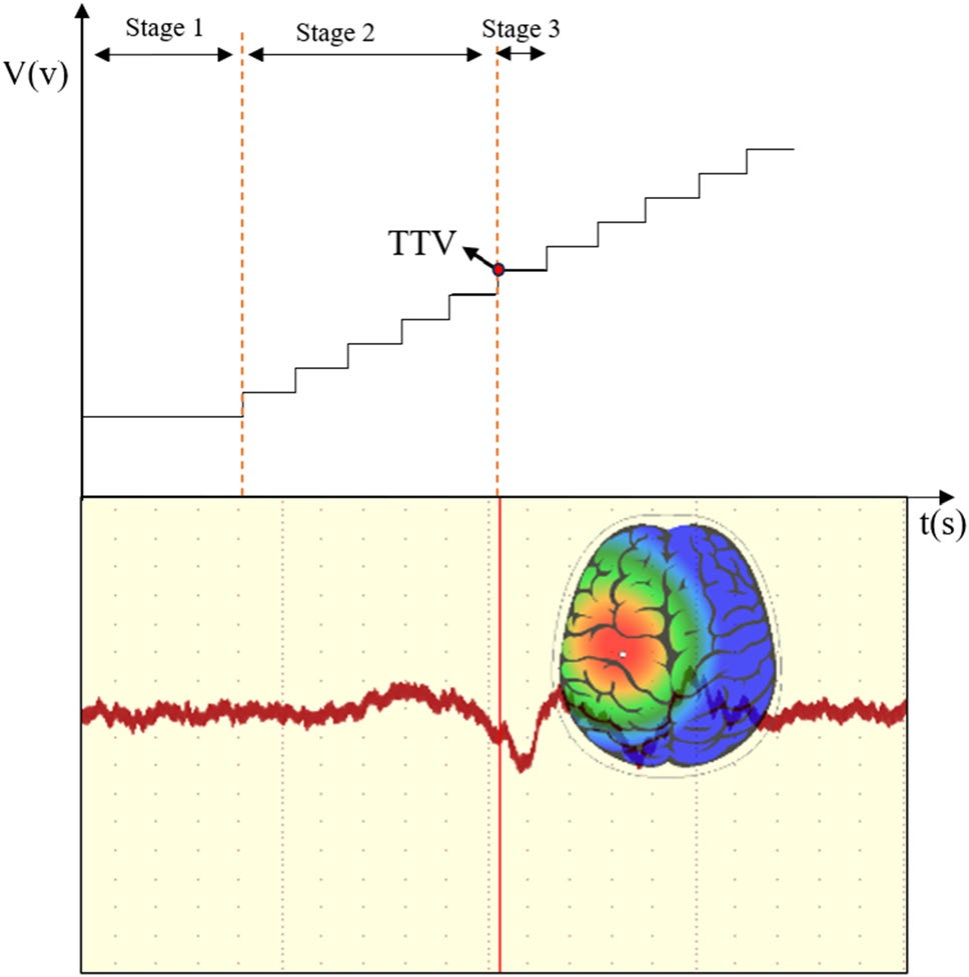
The schematic of TTV measurement. The voltage of the electrical stimulation increases gradually. When the voltage reaches a certain intensity, the C3 area of the cerebral cortex is activated, and this voltage intensity of the corresponding electrical stimulation is TTV. Stage 1: A 5-min standing adaptation before each measurement begins. Stage 2: The cerebral cortex has not responded yet with the gradual increase in voltage of electrical impulse device, the conditions (white noise and control) are presented in random order during the electrical stimulation. Stage 3: The voltage of the electrical stimulation reaches the TTV, and the cerebral cortex responds.

The perception of WNS was lower than participants’ tactile threshold, and it was difficult for participants to perceive white noise vibration themselves, and we would not inform participants whether to turn on white noise insole. We also ensure consistency in the experimental steps, and each participant needed to undergo a 5-min standing adaptation before each task begins. In addition, to reduce the degree of dispersion of the data, our measurement in this study were participants’ right feet and the interval between each measurement was set to 20 min. Figure 5 showed the schematic of TTV measurement.

### Statistical analysis

We used SPSS software (version 26.0, Chicago, IL, USA) for data analysis and processing. Given there were two sets of variables in the experiment, we used two-way repeated-measures ANOVA to compare the change in participants’ TTV. Levene’s test was used to analyze the homogeneity of the variances. The Kolmogorov–Smirnov test was used to evaluate the normality of the data. When the data were not normally distributed, Wilcoxon test was adopted. Statistical significance was accepted for values of p < 0.05. Tukey was applied for post-testing data. Cohen’s D was employed to measure the effect size (ES).

### Ethical statements

The human protocols used in this work were evaluated and approved by the Ethical Committee (HIRB) of Huashan Hospital Affiliated to Fudan University (Ethical Review No. 2019-059).

## Results

**Figure 6:**
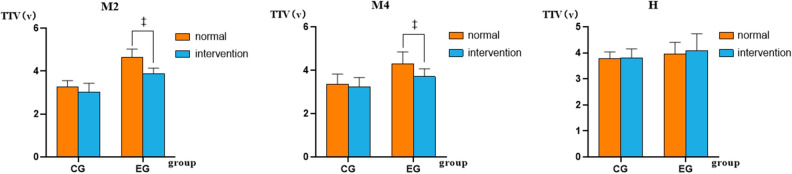
Comparison of TTV in M2, M4 and H areas. ^‡^Significant difference found between normal and intervention tasks. M2 = 2nd metatarsal, M2 = 4th metatarsal, H = heel, normal: without WNS intervention, intervention: WNS intervention.

We analyzed the mean values of TTV in the M2, M4, and H areas in right foot of CG and EG, as shown in Table 2. And Fig. 6 illustrates a detailed comparison of TTV in significant areas.

**Table 2:** Comparison of TTV between CG and EG.

		CG								EG							
M2*																	
Normal^†^	Right foot	3.09	3.25	3.39	3.37	3.27	2.71	3.36	3.70	4.40	4.61	4.8	4.78	4.65	3.88	4.76	5.23
	Average	3.27 ± 0.28								4.64 ± 0.39^‡^							
Intervention^†^	Right foot	2.95	2.83	3.27	3.55	2.51	3.02	2.51	3.52	3.66	4.23	3.56	3.81	3.99	3.58	4.11	4.12
	Average	3.02 ± 0.41								3.88 ± 0.26^‡^							
M4																	
Normal^†^	Right foot	3.66	3.25	3.64	3.78	3.94	2.89	2.82	2.78	4.28	3.74	5.34	4.51	4.30	4.38	3.44	4.30
	Average	3.35 ± 0.47								4.29 ± 0.56^‡^							
Intervention^†^	Right foot	3.17	3.84	2.75	3.12	3.04	3.82	2.70	3.41	4.11	4.02	3.34	3.39	3.9	3.55	4.05	3.24
	Average	3.23 ± 0.43								3.70 ± 0.36^‡^							
H																	
Normal	Right foot	3.62	3.75	3.88	3.87	3.78	3.27	3.86	4.17	4.42	4.13	4.15	3.02	3.76	3.70	4.10	4.29
	Average	3.78 ± 0.26								3.95 ± 0.45							
Intervention	Right foot	3.24	4.12	4.36	3.81	3.49	3.81	3.81	3.88	3.04	4.37	3.74	3.69	3.77	4.55	4.98	4.61
	Average	3.81 ± 0.34								4.09 ± 0.64							

M2 = 2nd metatarsal, M2 = 4th metatarsal, H = heel, normal: without WNS intervention, intervention: WNS intervention. *p* < 0.05.

*Interaction found between tasks and groups. ^†^Significant difference found between CG and EG. ^‡^Significant difference found between normal and intervention tasks.

The interaction with significance between tasks and groups was found in M2 area (p = 0.04). Simple main effects of tasks showed that TTV of the M2 in intervention task was significantly smaller than that in normal task in EG (p < 0.001, ES = 0.75). Simple main effects of groups showed that TTV of the M2 in CG was significantly smaller than EG in normal task (p < 0.001, ES = 0.90) and intervention task (p < 0.001, ES = 0.78).

There was no interaction between tasks and groups in TTV of the M4 and H area. In M4 area, significance of TTV was found between CG and EG in normal task (p < 0.01, ES = 0.67) and intervention task (p = 0.03, ES = 0.51). And in EG, TTV in intervention task was significantly smaller than that in normal task ((p = 0.04, ES = 0.53).

However, there was no significance of TTV found between CG and EG in normal task and intervention task in H area. And the application of WNS showed no evident effects on foot.

## Discussion

This study investigated the changes in the tactile thresholds of different external interventions in the main plantar pressure-bearing area of participants when wearing white noise vibration insole. During the experiment, the tactile threshold was redefined in the form of TTV. When TTV increased, it meant that the tactile threshold increases and the tactile sensing ability decreased; Otherwise, it meant that the tactile threshold decreased and the tactile sensing ability increased. As stated in the previous hypothesis, the TTV of EG was significantly reduced and tactile sensing ability was improved to varying degrees after the application of WNS.

Firstly, we needed to discuss the difference between mechanical stimulation (such as microfilament or vibratory testing) and electrical stimulation before conducting the experiment. Both mechanical stimulation and electrical stimulation were experimental means of simulating external stimuli. Mechanical stimulation involved the use of physical devices or instruments to stimulate the sensory system. Electrical stimulation involved applying electrical current to the skin or nerve endings to stimulate the sensory nervous system^21^. However, electrical stimulation and mechanical stimulation shared similar characteristics in providing external stimuli. A single effective electrical pulse could depolarize a nerve fiber once, and mechanical stimulation also induced synchronous activity in nerves. Talbot et al. mentioned that within the vibration period of mechanical stimulation, nerve fibers could be depolarized one to two time^22^. Vibol Yem et al. compared the sensation of electrical stimulation and mechanical stimulation intensity, and the results indicated that when the stimulation intensity was low, the sensation from electrical stimulation and mechanical stimulation was comparable. And when the pulse frequency of the electrical stimulation device was set to 30 Hz, the perceived intensity of electrical stimulation was comparable to the self-matched intensity of mechanical stimulation^15^. Therefore, under conditions of low intensity and appropriate pulse frequency, the difference between electrical stimulation and mechanical stimulation were not significant, making electrical stimulation may be a feasible method for detecting tactile thresholds.

A meaningful phenomenon appeared during the experiment. When electrical stimulation occurred, the fluctuation of the objective participants’ EEG was always ahead of participants’ active response to stimulation, which laterally reflected that the objective measurement of EEG was more sensitive and accurate^17^. This phenomenon could be speculated that active responses were generated by the brain, and the brain’s commands were issued after the EEG waveform, which was different from the classic knee-jerk reflex (a feedback action that could be completed without the involvement of the brain). Also, EEG detected brain activity by measuring the electrical signals of neurons in the brain, which took time to travel to the electrodes on the scalp for recording and analysis^23^. On the other hand, the body's response was transmitted and processed by the nervous system, including sensory information and motor commands, which were relatively fast but still required some time^23^. Specifically, the speed of sensory nerve transmission in the human body was about 10 m per second, which was relatively slow compared to the recording speed of EEG. The speed of EEG was usually in the millisecond range, while the response speed of the human body is usually in the range of hundreds of milliseconds to seconds^20^.

The lack of significant changes of TTV in CG may be due to the presence of tactile feedback in the healthy organism itself, such that it was difficult for the homophonic resonance of WNS to form an effective positive feedback mechanism on it, and inappropriate WNS intervention in individual areas could cause some degree of negative interference with TTV. However, TTV decreased significantly in EG at intervention task, we found that WNS improved the tactile sensation in M2 and M4 areas, as shown in M2 part and M4 part, in Fig. 6. It was speculated that the abnormal tactile feedback of the human body was unable to make reasonable adaptive adjustment to the distribution of plantar pressure, since the forefoot owned a larger plantar pressure, and the plantar pressure in M2 and M4 areas was relatively high in the proportion of the entire plantar pressure^13,24^. The forefoot had little subcutaneous fat and thin skin, once pressure was applied, local tissues were easily damaged and ulcers were formed^25^. Also, the forefoot assumed an important supporting role in walking, which could easily induce the formation of ulcers with the plantar pressure increased^26^. Therefore, the above two areas may be susceptible to occurrence of diabetic foot under frequent abnormal gait patterns. Above inference was also laterally confirmed in previous studies, the most common site for diabetic foot ulcers was the forefoot area^27^. The application of WNS lowered the tactile threshold of EG and improved plantar tactile feedback, participants were able to perceive subtle changes in the plantar area more acutely, thus reducing the incidence of foot injuries.

Further analysis compared TTV of plantar areas in EG between normal and intervention tasks, and the potential effects of WNS appeared to be limited in H area. This phenomenon could be speculated that variability in the density of plantar blood vessel distribution. The ulcer of diabetic foot was the result of vascular and neurological lesions in the foot^28^. Due to the reduction in blood flow to the lower limbs, the amount of blood oxygen and nutrients available to the foot was insufficient, then resulting in functional necrosis of the foot^29^. Previous study pointed that foot ulcers with vascular lesions took significantly longer to heal than foot ulcers of the same magnitude without vascular lesions, and the heel ulcers were the most difficult to heal in plantar areas^30^. It could be revealed that H area of the foot had a lower vascular density compared to the forefoot area in the physiology of the human body and therefore exhibit fewer negative effects in the progression of diabetic foot. Moreover, because the plantar pressure in H area was not as high as the forefoot area, the difference in plantar pressure in the H area led to a lower risk of injury during daily gait activities. The result suggested that the effect of WNS intervention in H area was not evident and the change of TTV may be related to the distribution of the plantar vessels and the pressure in the plantar areas. In response to the limitations of WNS in H area, measures to prevent damage in this area should focus on the correction of pressure. Rationalization of plantar pressure could help reduce the occurrence of injury and achieved active protection.

In this study, we used WNS to enhance the plantar sensation. Therefore, the applicability of artificially evoked sensation and natural touch was also worth contemplation. Artificially evoked sensation typically refers to stimulating nerves directly through external means to evoke sensory perception, while natural touch can be understood as the body perceiving external information such as force, shape, and temperature through the sensory receptors in the skin itself^31^. Therefore, the difference between artificially evoked sensation and natural touch lies in the different modes of stimulation of the receptors. The former was achieved through external means of stimulation, while the latter was achieved through the sensory receptors in the skin itself. Compared to the singular stimulation of artificially evoked sensation, natural touch stimulates multiple sensory perceptions, which could better activate neural circuits related to tactile sensation in the brain, thereby allowing participants to perceive tactile stimuli more accurately^32^. The advantage of artificially evoked sensation lies in its ability to directly stimulate nerves, minimizing experimental errors caused by variations in the condition of the plantar sensory receptors among participants in EG and CG^32^. Due to severe inhibition of foot sensory perception in the participants of EG, their ability to perceive external stimuli through their own sensory receptors was limited. Therefore, we aimed to improve participants' perception of the external environment through intervention with WNS at the beginning of the study. The short-term result also indicated that certain areas of the plantar surface in the EG showed significant improvement before and after the intervention with WNS. The effect of artificially evoked sensation far surpassed the damaged natural touch sensation. It could be revealed that, the sensitivity to direct neural stimulation was increased more than the sensitivity to natural touch sensation under short-term intervention of WNS.

Our study had several limitations. First of all, due to the small number of experiments and limited simulation scene (only in a static standing state). In the follow-up study, we will recruit more participants with diabetic feet and add some dynamic walking tasks to explore and analyze. Secondly, during the measurement process, EEG detection was so sensitive that unnecessary EEG signal fluctuations interfere with the test results sometimes. During the experiment, it was necessary to ensure that participants remain calm. Thirdly, the hasty use of electrical stimulation as a sensory testing method in this study may result in some bias in results, further exploration was needed to understand the difference between electrical stimulation and real sensory stimulation. Finally, since the self-developed insole was in the preliminary verification stage, there may be some deviations: one issue that was worth discussing is the setting of the frequency of the white noise vibration signal. Whether the frequency affected the final data results and the changes in tactile threshold was also a matter that deserved consideration. In addition, the size of insole was uniform, although the recruited participants had similar foot size, there may still be individual differences between each participant. In the future, we would consider making personalized custom insoles to ensure the fit for each participant.

## Conclusion

In summary, this study proved that WNS could be able to improve the plantar tactile sensing ability of participants with diabetic feet, but it did not cover all areas. WNS showed a potential positive impact on participants with diabetic feet in M2 area and M4 area, which lowered the tactile threshold in these areas. The influence of WNS on the plantar H area was still controversial, which was speculated that may be related to the difference in the distribution density of blood vessels in plantar areas. Due to the impaired natural touch in participants with diabetic foot, using artificial evoked sensation WNS intervention, would be a feasible approach to improve plantar sensation. We hope this work could contribute to the development of vibrating insoles for patients with diabetic feet.

## Data Availability

The data presented in this study are available on request from the corresponding author. The data are not publicly available due to privacy.
